# *Kingella kingae* Reveals Its Secrets

**DOI:** 10.3390/microorganisms10071261

**Published:** 2022-06-21

**Authors:** Pablo Yagupsky

**Affiliations:** Clinical Microbiology Laboratory, Soroka University Medical Center, Ben-Gurion University of the Negev, Beer-Sheva 84101, Israel; pyagupsky@gmail.com; Tel.: +972-506264359; Fax: +972-86403541

Sixty years ago, Elizabeth O. King, a bacteriologist working at the USA Centers for Disease Control (CDC), described a novel Gram-negative bacterium isolated from respiratory specimens, blood, bones, and joints. Based on its phenotypical features, the organism was initially allocated to the genus *Moraxella* and named *Moraxella kingii*, honoring King’s pioneering research [1]. In 1976, transformation experiments conducted by Henriksen and Bøvre, and meticulous studies of the biochemical properties and the fatty acid composition of the organism resulted in the allocation of the bacterium to a separate genus within the *Neisseriaceae* family, and it was renamed *Kingella kingae* [2].

For the first two decades following the initial description of the bacterium, *K. kingae* received little attention because it was thought to be an uncommon human pathogen, rarely recovered from patients with skeletal system infections or endocarditis [3]. Thus, the number of publications on the organism documented in the Pubmed database between 1960 and 1985 was only 26, and the niche of *K. kingae* in the human body, and the epidemiology and pathogenesis of the infections it caused remained unknown.

In 1988, the serendipitous discovery that the inoculation of synovial fluid aspirates obtained from young children with arthritis improved *K. kingae* isolation opened a new era in the study of the species [4] and disclosed its role as a common invasive pathogen during early childhood. The development of a selective blood-agar–vancomycin medium (BAV) inhibited the overgrowth of other members of the microbiota and facilitated the recovery of *K. kingae* in upper respiratory specimens [5]. The use of this tool revealed that the bacterium is a frequent component of the oropharyngeal flora of young children and allowed for the study of its interpersonal transmission among the healthy pediatric population [5,6]. A second quantum leap in the research of the organism and its diseases resulted from the use of nucleic acid amplification tests that enabled the detection of the difficult-to-culture *K. kingae* organisms, firmly establishing the species as the most common etiology of joint and bone infections in children aged 6–48 months, and reducing the fraction of culture-negative cases [7,8].

Following these exciting advances, interest in *K. kingae* grew exponentially, and the number of publications on the organism soared, reaching 65 in 2021 alone. In recent years, the intensive research has progressively widened our understanding of the organism and its virulence factors [9,10], the epidemiology of its carriage and spread in the young pediatric population [11,12], its peculiar clinical features [13,14], microbiological diagnosis [15], and antibiotic therapy [16].

This Special Issue, “*Kingella kingae*: Virulence Factors, Clinical Disease, and Diagnostics”, gathers five papers by an international group of renowned researchers who provide a timely update on this intriguing human pathogen and its diseases. The review article by Porsch [17] summarizes our current knowledge on the wide array of *K. kingae*’s constituents, which similar to other pathogens of respiratory origin, are responsible for the adherence of the bacterium to the oropharyngeal epithelium. Additional virulence factors facilitate bloodstream invasion and seeding to remote sites and ensure the survival of the organism in the skeletal system and endocardial tissues.

The article by Filipi et al., provides in-depth information on *K. kingae*’s Repeat-in-Toxin (the RtxA toxin), and its similarities and differences with other better-known members of the RTX superfamily of cytotoxins found in many Gram-negative organisms [18]. This secreted *K. kingae* component shows a wide range of deleterious activities affecting epithelial and phagocytic cells and chondrocytes and making it possible to colonize and break the respiratory epithelium, the bacterial circulation in the bloodstream, and the invasion of deep body sites [19].

The review by Yagupsky discusses our current understanding of the dual role played by the asymptomatic *K. kingae* colonization of the oropharyngeal mucosa as the source of hematogenous dissemination of the organism and its person-to-person transmission among the susceptible young pediatric population [20].

The article by Basmaci et al., explores the temporal association between viral infections, especially those affecting the upper respiratory and buccal epithelial surfaces, with invasive *K. kingae* disease [21]. These findings strongly suggest that the mucosal damage induced by a wide variety of viruses facilitates the translocation of the bacterium from the oropharynx, where its presence is innocuous, to the bloodstream, from which it spreads to skeletal tissues and the endocardium, causing invasive disease [21].

De Marco et al., provide a perspective on clinical *K. kingae* infections from the point of view of the pediatric orthopedist [22]. The invasion of the skeletal system by *K. kingae* is characterized by an atypical and unimpressive presentation, requiring a high index of clinical acumen. The diagnostic difficulty is aggravated by the frequent failure of routine culture methods to detect the organism in skeletal system exudates, necessitating the use of sensitive nucleic acid amplification tests.

The research article by Gouveia et al. [23] reports the results of a Portuguese study in which the demographic, clinical, and laboratory features of *K. kingae* septic arthritis were compared with those of joint infections caused by traditional pyogenic bacteria. The results clearly showed that *K. kingae* disease has a distinctive presentation characterized by younger age, low or absent fever, good general condition, low inflammatory markers, and an overall good prognosis.

Altogether, this Special Issue presents a broad panoramic view of our current understanding of the virulence factors and epidemiology of carriage of this emerging human pathogen, as well as the pathogenesis, clinical presentation, detection, and therapy of the diseases it causes. I hope that its contents will be of interest for clinical microbiologists, pediatricians, orthopedists, and infectious diseases specialists involved in the diagnosis and management of invasive infections in young children.

I thank all of the authors and reviewers for their valuable time and contributions that made this publication possible.
